# Contrasting patterns in phylogenetic and biogeographic factories of invasive grasses (Poaceae) across the globe

**DOI:** 10.1038/s44185-023-00016-4

**Published:** 2023-05-18

**Authors:** Luis R. Pertierra, Pablo A. Martínez, Juan G. Rubalcaba, David M. Richardson, Miguel A. Olalla-Tárraga

**Affiliations:** 1 BIOMA Lab, Departamento de Biología, Geología, Química y Física Inorgánica, Universidad Rey Juan Carlos, Móstoles, Spain; 2 Department of Plant & Soil Sciences, University of Pretoria, Pretoria, South Africa; 3 PIBi Lab, Departamento de Biologia, Universidade Federal do Sergipe, São Cristovão, Brazil; 4 Department of Botany & Zoology, Centre for Invasion Biology, Stellenbosch University, Stellenbosch, South Africa; 5 Department of Invasion Ecology, Institute of Botany, Czech Academy of Sciences, Průhonice, Czech Republic

**Keywords:** Biogeography, Grassland ecology, Invasive species

## Abstract

Grasses (Family Poaceae) are among the most successful invasive plants in the world. Here we evaluate phylogenetic and biogeographic patterns of emergence of naturalized and invasive species among grasses globally. In our data, circa 19% of the grasses are currently catalogued as invasive and almost 38% are listed as naturalized; these are among the highest ratios for single families of organisms. Remarkably, most tribes of grasses contain numerous naturalized and invasive species, suggesting that the invasion success is rooted broadly in ancestral traits in the Poaceae. Moreover, the probability of invasiveness is positively related to the diversification rates in the family also suggesting a link with recent radiation events. The phylogenetic distribution of the invasive condition is neither strongly conserved nor purely random. Phylogenetic clumping levels also vary between Poaceae subclades. We postulate that this diffuse clumping could be partially attributed to the expression of labile traits that contribute to species invasiveness. In addition, floristic regions (biomes and biogeographic realms) have different proportions of invasive species, with the temperate Palearctic region having the highest ratio of invasive vs. non-invasive species. The phylodiversity of aliens across regions is also variable in space. Comparison of alien phylodiversity levels across biogeographic realms and biomes reveals regions producing highly restricted invasive lineages and others where the diversity of aliens exported is no different from global mean diversity levels in grasses. Elucidating the evolutionary patterns and drivers of invasiveness is useful for understanding and managing invasions, with the low phylogenetic structure of alien grasses warning of their overall high invasiveness potential.

## Introduction

Thousands of invasive species contribute to the homogenizing and altering of ecosystems around the world in the era of the Anthropocene^1^. Biological invasions are widely recognized as a major contributor to biodiversity loss, ecosystem degradation and deterioration of ecosystem services^2,3^. In this context, the drivers and factors that determine competitive success in biological invasion processes have been widely studied^4^. We know of no studies, however, that have systematically explored the phylogenetic and biogeographical patterns of emergence among all invasive species globally for a given monophyletic plant group. Nevertheless, several continental, regional and local area-specific studies have produced insights that hint at the existence of structured patterns globally^5,6^. Moreover, while relatedness among the pools of alien species remains largely underexplored, the relatedness between introduced alien and recipient native flora been examined for some groups and shown to decrease along the introduction–naturalization–invasion continuum^7^. Darwin’s naturalization conundrum postulates the trade-off in the competitive gains for aliens in having trait singularity (novel weapons) to thrive in a new environment, against sharing adaptive similarities with natives in order to persist in it^7^. This question represents a promising aspect of invasion science to study further from an evolutionary perspective. In this context, Poaceae stands out as an obvious model group. Poaceae is one of the best producers of alien invaders globally; invasive success in this family has been widely attributed to the combination of different dispersal, establishment and other ecological traits^8^. Understanding the invasive patterns among Poaceae taxa could provide unique general insights into the broad phylogenetic conservation structure of biological invasions and inform the development of pre-emptive biosecurity risk assessments that can screen for latent species of high invasive potential.

The question of phylogenetic signal (also referred to as phylogenetic clumping), i.e. the tendency for closely related species to share similar traits or conditions is often a matter of ecological interest in plant science^9,10^. However, the phylogenetic signal of species invasiveness has been studied in only a few groups such as avifauna^11^. Interestingly, recent studies found weak linkages between adventive range size (a measure of invasion success) and phylogenetic ancestry among invasive birds^12^. This indicates that the invasive potential of birds is strongly inherent in species and not widely conserved among close relatives. It is intriguing to examine whether grasses as a whole also show such weak phylogenetic structuring. Poaceae has a high number of alien species, being a highly successful “factory” of aliens, but internally the emergence levels may vary. Consequently, from here on we refer to those lineages that tend to concentrate alien species as “phylogenetic factories of invasive species”. Moreover, it has been postulated that invasiveness may relate to the diversification rates of the evolutionary lineages themselves by favouring an increased source pool of diversity from where some species can have the traits to thrive in the Anthropocene^13^; this key hypothesis remains to be tested empirically and is also examined here.

Poaceae has distinctive subgroups which could hypothetically host different levels of phylogenetic clustering^14,15^. Two major sister lineages are classically recognized: the BOP (comprising Bambusoideae, Oryzoideae, and Pooideae) and the PACMAD (comprising Panicoideae, Arundinoideae, Chloridoideae, Micrairoideae, Aristidoideae, and Danthonioideae) grasses^16^. BOP grasses use the C_3_ photosynthetic pathway and are typically present in temperate and cold regions. They comprise three distinct subfamilies, the cosmopolitan Oryzoideae (cultivated rice and wild relatives), the low latitudinal Bambusoideae (bamboos), and the predominantly “cool-season” Pooideae (typically comprising lawn and highland pasture grasses). In turn, PACMAD grasses (typically comprising cereal crops and savanna grasses) have complementarily developed the C_4_ photosynthetic pathway. PACMAD lineages are typically present and intermixed in grasslands of temperate and tropical regions.

Lastly, the diversity of invasive species can also be shaped by the biogeographical origin of the plants. Regional conditions can promote invasive traits across the suite of species in an area, regardless of the phylogenetic lineages. Thus, it can be expected that some parts of the globe have invasive species with more evolutionary affinity than others. Consequently, we refer to those regions where large numbers of aliens emerge from distantly-related groups as “biogeographical factories of invasive species”.

Considering these evolutionary and biogeographic factors, the invasive clumping among Poaceae lineages would hypothetically be affected by the dominant drivers for invasiveness in each factory group (phylogenetic source pool) and factory region (biogeographic source pool).

## Testing invasion hypotheses

The rich dataset and structure of invasive grasses can be used to test for various invasion hypotheses linked to (1) phylogeny or (2) biogeography (summarized in Table 1).

**Table 1:** Summary list of newly examined invasion science hypothesis tested for (1) phylogenetic patterns and (2) biogeographical patterns.

Test	Hypothesis	Outcomes	Interpretation
(1) *Phylogenetic patterns of invasive species*			
1.1 Phylosignal with PhyloD	Random evolution	*D* = 1	Purely random emergence
	Brownian/Neutral evolution	*D* = 0	Strong neutral clumping
	Neither Random nor Brownian	1 > *D* > 1	Moderate relatedness
	Null	1 = *D* = 1	Inconclusive
1.2 Diversification rates	Diversification alien promotion	% ~ Div.	Rate of evolution favour invasiveness emergence
	Null	% × Div.	No relationship
(2) *Biogeographical patterns of invasive species*			
Phylodiversity with SES PD	Generalized regional invasiveness	PD invas = PD all	Ample regional alien diversity produced
	Restricted lineage invasiveness	PD invas < PD all	Reduced regional alien diversity produced

### Examination of patterns of phylogenetic factories of invasive species

Sister plant species with similar life histories may share common invasive trait adaptations (e.g. reduced seed mass for wider human-assisted dispersal or higher specific leaf area for faster opportunistic growth)^17^. Thus, a strong invasive trait preservation would lead to the observation of a significant phylogenetic structure of invasiveness (seen as phylogenetic clumping). Early studies already raised the question of the phylogenetic relatedness of invasive species and documented different levels of invasiveness among vascular plant families^18,19^. However, to date, it remains to be confirmed whether the invasive status of species tends to be phylogenetically conserved. Importantly, a previous study suggested that phylogenetic signals are scale-dependent^5^, and that strong evolutionary conservatism is evident at a continental scale but less so at a landscape scale. Because most evolutionary invasiveness studies have been conducted at continental and local scales, the extent to which the relatedness of invasive species is phylogenetically structured at a global scale remains largely unexplored. Here, the invasive success can be attributed to the confluence of different traits with different phylogenetic clumping (i.e., degree of phylogenetic conservationism)^11^, with some of them hypothetically relevant to all regions. For instance, in aquatic plants allelopathic potential related to invasive capabilities has shown a strong phylogenetic signal^20^.

#### Evolutionary pattern of the invasive status among Poaceae and major subclades

We investigate the phylogenetic patterns of naturalization and invasive status as potentially inheritable traits among the Poaceae family by testing two alternative (non-mutually exclusive) hypotheses with four alternate scenarios:

##### Random emergence (no clumping)

First, a purely random association of invasiveness across the phylogeny (no clumping) would mean that the invasive status is not driven by traits that have been conserved among closely related species. In this scenario, more evolutionarily labile traits would be responsible for invasive success.

##### Neutral emergence (evolutionary clumping)

In parallel, a phylogenetic clumping pattern consistent with Brownian inheritance (sensu lato) as a model of strong evolutionary association would indicate that invasiveness is ultimately determined by conserved biological traits^17^ that can lead to strong aggregations or “patches” of alien species in the phylogeny.

##### Neither random nor neutral clumping

If both previous hypotheses are rejected then intermediate levels of phylogenetic clumping are observed. This would indicate that both phylogenetically conserved and intrinsically labile features of species are implicated in the determination of the level of invasiveness^21^.

##### Inconclusive evidence of evolutionary clumping

Lastly, if both hypotheses cannot be discarded, there is no evidence to reject that the association is neither clumped nor random and there is thus no support to infer any structure of evolutionary relatedness.

#### Effect of diversification rates in the invasiveness per genera

The rapid diversification of grasses has been postulated as a reason for high levels of invasiveness within the order Poales^8^. This leads to the question of whether this effect is merely a general feature of grass diversity overall (simply being more species-rich than other families), or whether the evolution patterns also play a role in the invasiveness at a finer taxonomic resolution. Specifically, we examine whether the diversification rates correlate with the presence of invasive species after controlling for their relative species richness. A significant relationship would thus indicate that diversification plays a role in finer taxonomic units. We would expect to observe this relationship, as naturalization and invasion are recent phenomena relative to the evolutionary age of grasses, which have a short history of association with humans. Thus, it is plausible that grass invasiveness has been enhanced by recent past diversification events, making some species particularly well-suited to thrive in the Anthropocene. We refer to this as the “diversification-favoured invasion hypothesis”.

### Examination of patterns of biogeographical factories of invasive species

Biogeographic filters can also uncover additional clues pertaining to patterns and processes implicated in the emergence of invasiveness levels and evolutionary selection. Importantly, invasiveness has also been linked to evolutionary processes concentrated in certain regions of the world where environment and evolutionary history have interacted to generate a flora that is particularly adapted for survival, persistence, growth, proliferation and the capacity to spread rapidly. For instance, a relatively high number of grasses originating in southern African arid savannas have become widely invasive^22,23^. In this situation, abiotic environmental and/or biotic competitive filters may generate disproportional invasiveness clumping from specific lineages in certain regions.

This leads to the question of the relative phylogenetic diversity and contribution of invasive Poaceae species per floristic region against the global Poaceae pool of diversity. The spectrum of life histories of grasses and forms of influence from the human activity in each factory region could result in high alien diversity (e.g. a generalized promotion of human association) that is indistinguishable from the general pool of diversity; we refer to this as the “generalized invasiveness promotion hypothesis”. This scenario is met when the observed phylogenetic diversity (PD) measured in the form of a species-richness pooled Faith’s PD is no lower than the null score^24^, meaning that the species are locally less related (no less diverse) than the global average. Alternatively, an observed low PD can also occur (e.g. by stronger promotion of certain lineages). We refer to this effect as the “restrictive lineage invasiveness promotion hypothesis” where the observed PD value is significantly lower than the null score from the global average. Areas with no lower than random PD values of the invasive pool would have a broad range of equally successful invasive lineages to invade new regions; these regions can thus be considered biogeographical factories. In turn, source regions with comparatively low PD of their invasive species pool are donors of more phylogenetically-restricted lineages that are associated with invasiveness. These lineages can thus be identified as evolutionary factories that would also merit increased biosecurity attention.

In summary, we investigated the phylogenetic and biogeographic patterns of invasiveness among Poaceae to obtain insights into the evolutionary structure and the role of biogeographic regions in the emergence of invasive species to inform general invasion science and biosecurity management. Overall, the examination of phylogenetic signals and biogeographic patterns is deemed highly relevant for establishing causal traits responsible for phylogenetic paths (e.g. diversification). Understanding these phylogenetic processes also has practical applications in horticulture and ethnobotany^25^, agriculture and forestry management^26^ as well as in habitat restoration and wilderness conservation^27^.

## Results

### Poaceae invasive load

A total of 481 (~36%) naturalized alien species (NAS) and 246 (~19%) invasive alien species (IAS) were identified from a total of 1319 species present in Qian and Jin’s 2016 phylogeny^28^ (see Table S2).

### Evolutionary pattern of the invasive status among Poaceae and its major subclades

**Fig. 1:**
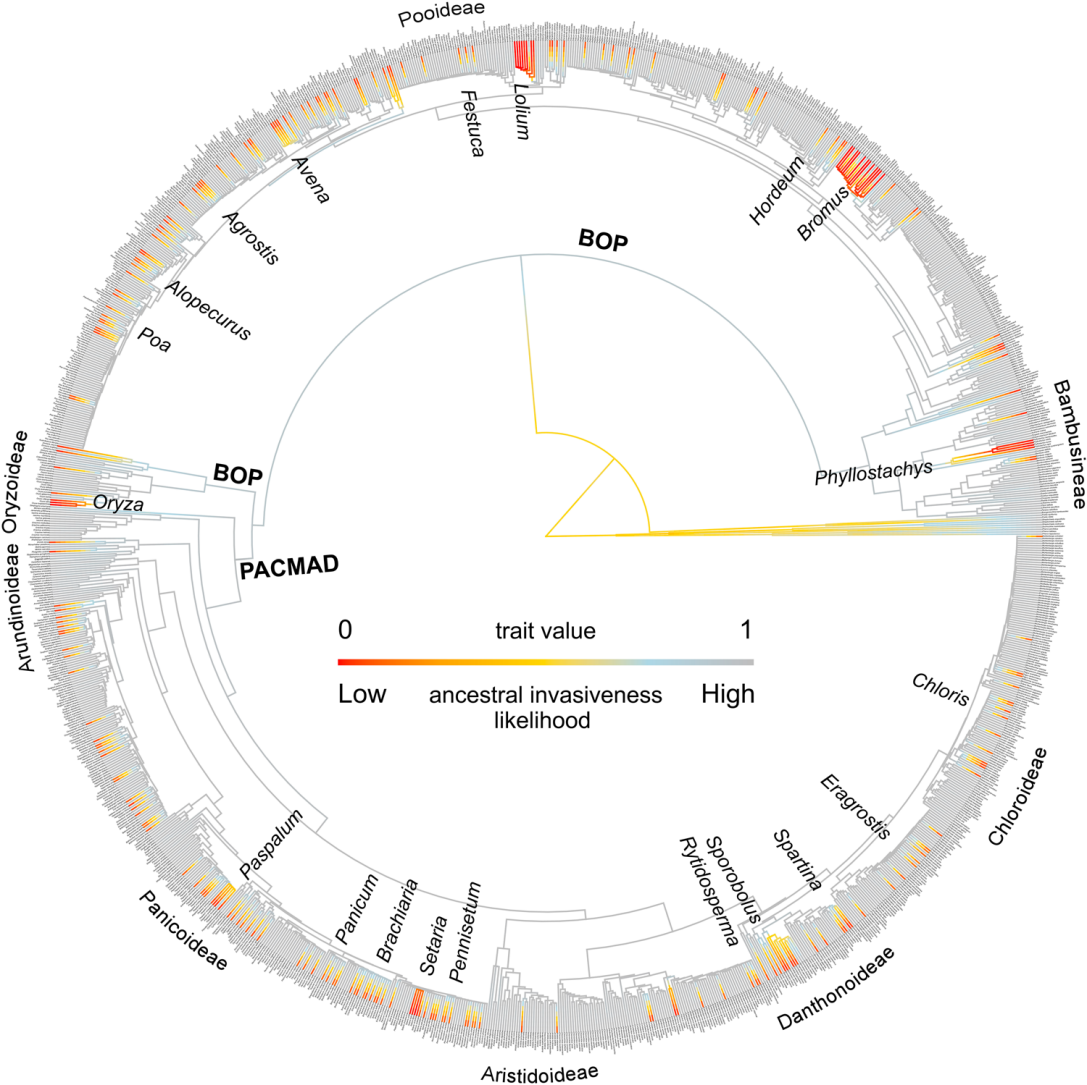
Mapping of the predicted invasiveness inheritance probabilities along the Poaceae family (alien invasive species are shown in red). Genera with a high number of invasive species are highlighted. A lambda transformation of 0.05 was done to enlarge the tips and assist its visualization. Due to this transformation (for visualization purposes), this graph has a small distortion and cannot be taken as a direct ancestral state reconstruction. An untransformed high-resolution file can be found in S3.

The phylogenetic tree fan graph shows the emergence of nodes of invasiveness within the family (Fig. 1). The main clusters of IAS observed in Fig. 1 correspond to the genera *Bromus* and *Lolium*. Interestingly, *Bromus*, which has the largest number of IAS, has one particular intrageneric clade with a high number of invasive species (12 out of 18), whilst IAS are nearly absent in the rest of the genus (only two out of 22 species) (see Fig. 1).

**Fig. 2:**
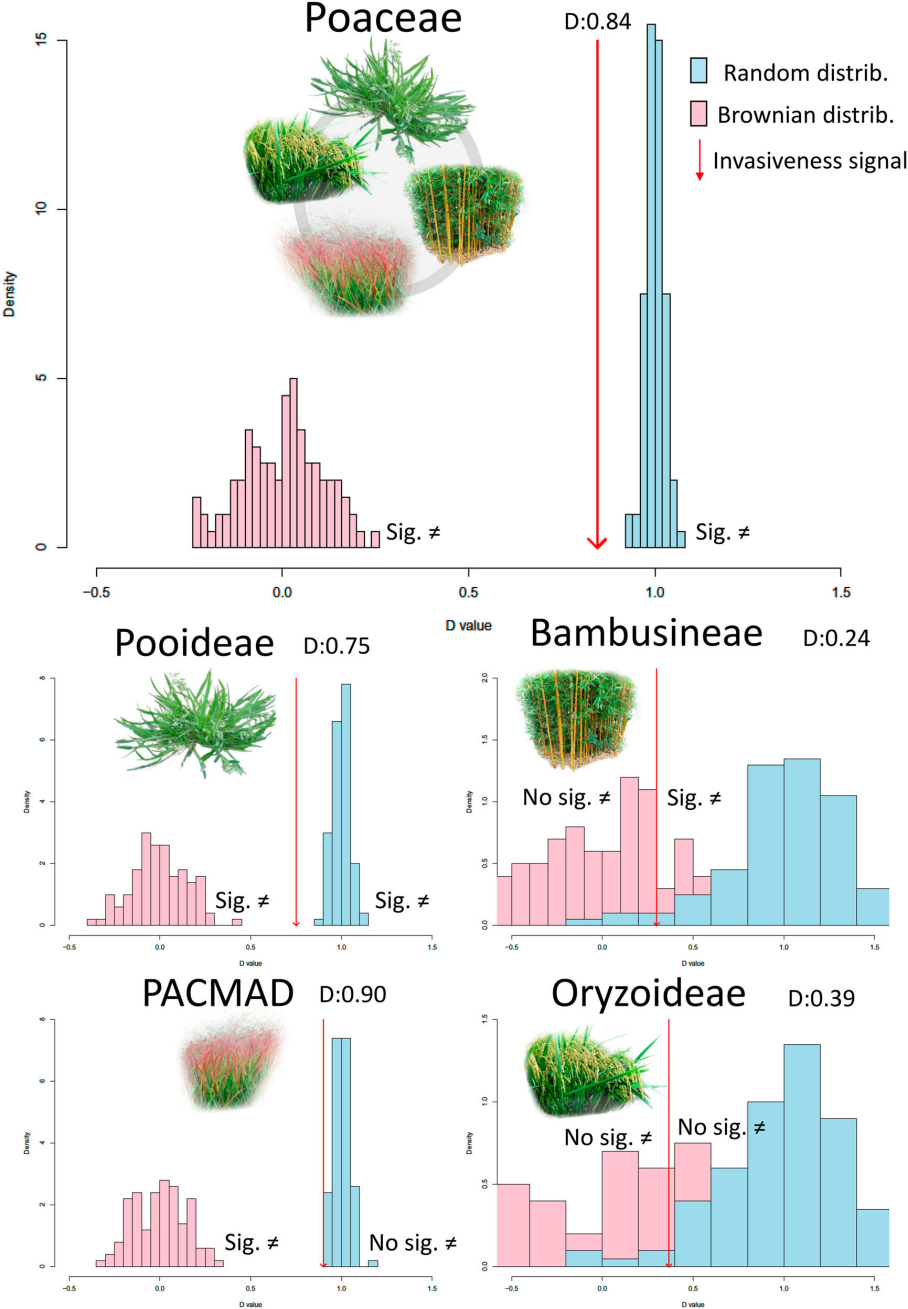
Graphic representation of PhyloD probability curves on the estimation for the phylogenetic clumping signal of the invasive character in Poaceae as a whole and within the four main Poaceae subclades: Pooideae, Bambusoideae, Oryzoideae and PACMAD. Expected Brownian inheritance distribution is shown in pink bars; Expected Random distribution is shown in blue bars. Red arrows indicate observed PhyloD:values. All silhuettes were photo edited from free usage images (https://creativecommons.org/).

We next analysed the dominant type of evolutionary processes that led to the general distribution of the invasive character across the phylogeny. The PhyloD value of 0.84 for the invasive character among the whole Poaceae phylogeny indicates that the clumping signal is significantly different from random occurrence (lower than one), while also greater than zero and thus not as strongly structured as would occur under a strong Brownian clumping (Fig. 2, Table 2). When looking by groups, the evolutionary signal for PACMAD is weakened to the point of being considered statistically random (PhyloD values close to 1) with no justification for rejecting a stochastic distribution (but note that the PhyloD levels, 0.90, are not much higher than the rest). In the case of Pooideae there is a moderately stronger phylogenetic structure (0.75) but this is still distinguishable from a strict Brownian character inheritance clumping (PhyloD values close to 0). In the case of Bambusoideae, there is no evidence to reject Brownian evolution for the invasive character (values are close to 0, Fig. 2), with the character being clustered around the genera *Bambusa* and *Phyllostachys*. In turn, in the case of Oryzoideae there is no evidence to reject any evolutionary model (PhyloD values are indistinctly close to both 1 and 0, Fig. 2). Lastly, no invasive species are found within the basal group sister to the rest of previous lineages. Interestingly, the global NAS dataset structure (0.72) is moderately stronger than the global IAS one (0.84) and so the signal appears to be more phylogenetically conserved across relatives at earlier stages.

**Table 2:** Evolutionary clumping (PhyloD) estimate o for the naturalized (a) and invasive character inheritance (b) among Poaceae.

Clade	Nat./Total	Phylo D	Brownian Evol.		Random Evol.	
(a) *Naturalized alien species*						
Poaceae (all)	479/1319	0.72	0*	Reject	0*	Reject
PACMAD	258/673	0.73	0*	Reject	0*	Reject
Pooideae	194/560	0.61	0*	Reject	0*	Reject
Bambusoideae	19/51	0.68	0.03*	Reject	0.02*	Reject
Oryzoideae	7/26	0.43	0.28	Evidence	0.11	Evidence
**Clade**	**Inv./Total**	**Phylo D**	**Brownian Evol.**		**Random Evol.**	
(b) *Invasive alien species*						
Poaceae (all)	246/1319	0.84	0*	Reject	0*	Reject
PACMAD	127/673	0.90	0*	Reject	0.09	Evidence
Pooideae	105/560	0.75	0*	Reject	0*	Reject
Bambusoideae	7/51	0.21	0.34	Evidence	0.02*	Reject
Oryzoideae	6/26	0.37	0.34	Evidence	0.08	Evidence

Values close to 0 indicate strong Brownian/Neutral evolutionary clumping, whereas values close to 1 indicate low phylogenetic clumping of the naturalized/invasive status in the data. PhyloD values not significantly different from 0 indicate a signal of Brownian/Neutral Evolution (strong clumping) and not significantly different from 1 indicate a strong signal of Random Evolution (no clumping) of traits leading to invasiveness or naturalization.

### Effect of diversification rates in the invasiveness per genera

Based on the global Poaceae dataset the diversification rates obtained with BAMM were examined. A positive, significant relationship between invasiveness to diversification rates was found (*β* = 1.18 ± 0.49, *z* = 2.43, *P*-value = 0.015; phylogenetic correlation *α* = 0.049).

### Biogeographical factories of invasive species (total and standardized values)

**Fig. 3:**
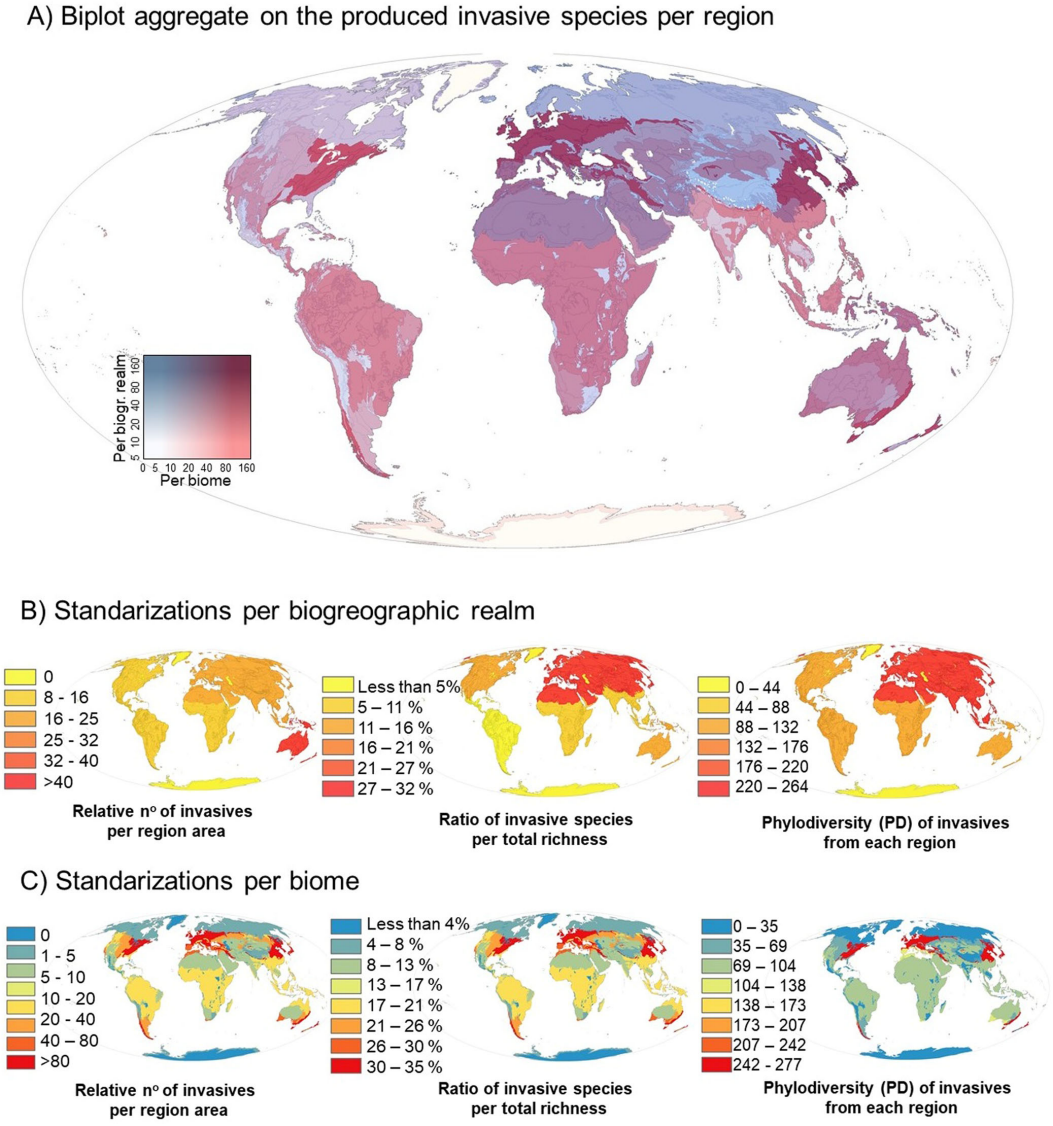
Biogeographic origins of invasive species of Poaceae. **A** Biplot display of invasive species generated per floristic region with the aggregated numbers of emergence by biogeographic realm (blue gradient) and biome (red gradient). The basemap of Earth was taken from ArcGIS-licensed software. Purple colours indicate high contribution in both terms, thus highlighting areas that act as important invasive species factories. Dark blue areas indicate weak biome contributors within alien-rich realm factories of invasives. Dark red areas indicate strong biome factories of invasives at poor realm contributors. **B**, **C** Standardized numbers of invasive species production per biome/realm factory size, per regional biome/realm richness of Poaceae species pool (in the phylogeny) and the accumulated total phylogenetic diversity (PD) of the invasive species community per biome/realm, respectively.

Figure 3A shows the overlay of invasive species originated per region (realm and biome combined biplot). Among IAS, the largest factories of grasses in total numbers per realm were the Palaearctic and Nearctic realms (Table 3). The Australasian and Afrotropical realms are placed at an intermediate level. The lowest contributors were the Indo-Malay and Neotropical realms. In terms of biomes (Table 4), the temperate broadleaf forest biome, comprising a range of natural and anthropized habitats at intermediate latitudes, contributed by far the highest number of IAS. At an intermediate level were the tropical moist forests biomes, Mediterranean shrublands, savannas and desert biomes. The lowest contributors of IAS were tropical dry and coniferous forest biomes, as well as taiga and montane grassland biome systems. Figure set B1 and C1 indicate the relativized number of aliens produced per region after adjusted for the area, Figure set B2 and C2 depict the ratio of aliens produced from the total of native species contained in the phylogeny studied. Figure set B3 and C3 display the total phylogenetic diversity (PD) richness of aliens produced per region. The ratios of naturalized/invasive to total species emergence and the total PD richness per each biogeographic realm and biome are shown in Tables 3 and 4, respectively.

**Table 3:** Total Phylogenetic Diversity richness (PD) per floristic factory realm of all the globally-listed alien grass species with respective invasive/naturalized status originated in each region.

Realm	Total factored species			Ratio of alien sps.		Phylogenetic diversity	
	Tot.	Nat.	Inv.	Nat.	Inv.	PDNat.	PDInv.
Palaearctic	378	193	122	51	32.3	339.1	263.7
Nearctic	245	109	26	44.4	10.9	236.7	114.4
Australasian	270	64	42	23.7	15.1	164.1	129.1
Afrotropical	150	45	23	30	15.3	121.1	78.2
Indomalay	57	33	17	57.9	29.8	113.1	87.6
Neotropical	120	35	15	29.2	12.5	104.6	41.2

**Table 4:** Total Phylogenetic Diversity richness (PD) per floristic factory biome of all the globally-listed alien grass species with respective invasive/naturalized status originated in each region.

Biome	Total factored species						Ratio of alien sps. (%)		Phylogenetic diversity	
	Tot.	Nat.				Inv.	Nat.	Inv.	PDNat.	PDInv.
Desert	126				42	11	33.3	8.7	112.2	71.9
Mediterranean shrub	137			63		25	46.0	18.2	173	120.4
Montane grassland	73		3			0	4.1	0	38.6	0
Taiga	6		3			2	23.1	15.3	13.7	4.3
Temp. broadleaf forest	393		211			138	53.4	35.1	383.5	277.7
Temp. coniferous forest	76	18				5	23.7	6.6	93.8	48.6
Temp. savanna & shrub	159	33				25	31.7	7.7	108.1	65.5
Trop. coniferous forest	19	2				0	10.5	0	0.5	0
Trop. dry forest	9	3				1	33.3	11.1	25.1	0
Trop. moist forest	125	61				29	48.8	23.2	164	96.7
Trop. savanna & shrub	159	40				25	25.1	15.7	94.6	71.6
Tundra	16	2				1	12.4	6.2	4.3	0

When controlling by the species pool accounted in the analyses per region the standardized (SES), PD values indicate that all realms follow the restrictive promotion hypothesis for naturalization, but only three of them for the invasiveness (Neotropical, Palaearctic and Afrotropical), with the least specific realm factory being the Nearctic and the most restrictive area the Neotropical realm. In turn, the levels of aggregation per biome of aliens are more variable. Six biomes fell under the restrictive promotion for naturalization, whereas five of them were generalized factories of aliens. In the case of biome, only three of them were restrictive, and the rest were unspecific factories. Excluding regions with only one invasive (see Table 4) temperate savannahs were the least specific biomes, leading to a generalized invasiveness promotion. In contrast, tropical savannah and tropical moist forest biomes contained the most lineage-restricted aliens.

## Discussion

### Poaceae invasive load

Our results confirm that grasses have a remarkable abundance of IAS, which is consistent with previous assessments^8^. Furthermore, the ratios of naturalized to non-naturalized invasive to non-invasive species in Poaceae are particularly high. The Global Naturalized Alien Flora database (GloNAF) repository estimated 3.9% of the global flora to be naturalized^1,29^; in the case of our list of phylogenetically resolved Poaceae we obtained a much higher ratio of naturalized species (37.8%) and a remarkable number of invasive species (around 19%). Still, our estimations of the invasive species ratio in Poaceae based on the phylogenetic dataset used may fall short given the larger number of species introductions. Nonetheless, not all introduced species worldwide are expected to become IAS^30^. Moreover, another consideration to bear in mind is that naturalized and invasive species are logically expected to be more present in resolved phylogenetic trees as they are often abundant in the environment and/or of particular interest. Hence, the proportion may be reduced if more complete trees with larger pools of species were available. Despite these limitations, this account provides, in our view, the best possible estimate of the relative abundance and ratio levels of NAS and IAS in a phylogenetically-resolved Poaceae phylogeny. Our analyses also consider source floristic regions at the biogeographic level, an approach that could be replicated for other taxonomic groups to reveal information on the relative pressure intensity of biological invasion that different clades and sites of origin pose worldwide^22^.

### Evolutionary pattern of the invasive status among Poaceae and its major subclades

Phylogenetic conservatism of the invasive character is moderate, being neither random nor strongly clumped (Fig. 2, Table 2), a pattern consistent with findings for birds^12^. A weak evolutionary clumping is not uncommon in the natural world; for instance, a similar result is observed in Primates where some behavioural traits also have weak phylogenetic structure^31^. Phylogenetic conservationism of fungal endophyte communities has been also found to drive host plant growth^32^. For the case of IAS, moderate levels of phylogenetic conservation were found. Only in the case of PACMAD was there sufficient evidence of a purely random emergence of IAS. In turn, we have some evidence of a strong clumping structure of invasiveness in BOP among the Bambusoideae, which could be potentially related to the strong selection of specific lineages for ornamental uses^33^. However, the small available sample size for this large and diverse group calls for caution in interpretation. Interestingly, the evolutionary structure at the level of NAS is slightly higher across all levels than the IAS, hinting that the traits operating for the species establishment are perhaps more conserved than those for outcompeting natives and altering ecosystem functioning. This result would support the postulate of Darwin’s naturalization conundrum of stronger lineage identity at the invasion end. However, since the NAS and IAS status listings come from different databases with different assignation criteria any interpretation around the invasion-stage-continuum should be made with caution. Here we restrict much of the specific hypothesis testing analyses and results interpretations to the invasive clumping signal (IAS dataset) for the sake of consistency.

The low evolutionary clumping of both the NAS and IAS datasets raises numerous questions. Overall, these results indicate a fine evolutionary selection within recent adaptive radiations towards selecting some ‘invasive-successful’ species among the existing pool of each lineage. These invasive species require traits not only to colonize but also to outcompete natives so as to thrive in new environments. However, the expression patterns of specific traits that make them uniquely invasive are yet to be disentangled from an evolutionary perspective. Moreover, although we see a relationship with diversification levels we still cannot immediately infer whether invasiveness is a direct probabilistic consequence of pre-existing natural diversity levels or evolutionary radiation of diversity consequence of anthropogenic pressures. The pattern of emergence of IAS is typically linked to those floral groups exapted to human activities and disturbances, often resulting from a long history of coexistence^34^. Thus, the high ratio and low evolutionary clumping of IAS in Poaceae could possibly be attributed to their evolutionary histories, where several “Old World” IAS grasses from very different evolutionary origins have been subjected to different human uses historically. This matches our spatial results where the Palaearctic is identified as the region producing the highest ratios of alien species (see Table 3), as well as Mediterranean and Temperate broadleaf forest regions (see Table 4) which are biomes frequently occupied and transformed by humans historically^35^. Cultivated species are recurrent examples causing some of the clusters observed in our phylogeny; while we found no reports of *Triticum* (wheat) species becoming invasive anywhere, several other crops such as *Avena* (oat)*, Oryza* (rice), *Hordeum* (barley), *Secale* (rye) and *Zea* (corn), have invaded other parts of the world. In turn, species used for fodder are widely dispersed and have become invasive (e.g. *Eragrostis*, *Festuca*, *Sorghum*) showing some prominent clusters in our phylogeny (Fig. 1). In this regard, *Bromus* has a particularly high number of invasive species, as several of them have benefited greatly from their usage as pasture grasses for cattle. The same applies to *Lolium* species, with the six species in the phylogeny being considered invasive, including the less widespread *Lolium persicum*, which has been listed as invasive in China^36^. Alternatively, some IAS benefit from their adaptation to human activities as inadvertently transported weeds. Invasive success relates to broad climatic tolerance, wider dispersal capabilities and fast reproduction. For example, *Poa annua* and *P. pratensis* are two of the most highly invasive species worldwide thanks to such versatility, being present on all continents including Antarctica^37^. In other cases, the growth of non-native lawn grasses is specifically promoted by humans due to their invasive capabilities such as aggressive sod and turf-forming plants (e.g. *Agrostis stolonifera* in golf courses). Lastly, some clusters derive from the use of sister species as ornamentals (e.g. bamboo from the genus *Phyllostachys*)^25^ with the current risk of additional planted species of commercial interest becoming invasive^38^. Overall, all major groups of grasses have large numbers of IAS but present remarkably different levels of phylogenetic clumping.

The biogeographic patterns and carbon specialization traits of the two major clades (BOP (specifically, Pooidae) and PACMAD) hint at the existence of spatial and/or evolutionary effects acting in modulating the strength of the phylogenetic signal, but not the frequency of IAS emergence. The PACMAD complex (dominated by hot and dry grassland tolerant species) and the BOP Pooideae (composed mainly of frost-hardy grassland species) are naturally exapted to dominate different environments (tropical-temperate versus cold regions)^16^. Interestingly, Pooideae had a stronger (but possibly anecdotal) phylogenetic signal compared to the PACMAD complex. From this, we could speculate that IAS within cold-tolerant groups such as the “cold-season grasses” would be more intensively driven by conservative forces (hypothetically, thermal niche limits and breadth), whereas the emergence of IAS in temperate-tropical dominant groups such as PACMAD could be driven by more labile drivers such as competitive traits. However, the PD study of the respective pools of alien species per realms and biomes did not show a consistent pattern with the carbon specialization hypothesis. Deeper examination of the trait drivers behind the observed higher invasiveness conservationism among BOP grasses is thus required.

The observed high IAS ratios in temperate regions could be partly explained by the thermal centrality of their niches, making them well-equipped to colonize more environmental ranges (southern or northern expansions). In turn, biomes of environmental extremes produce lower invasive species numbers, yet without suggesting any form of a latitudinal pattern of standardized PD levels. The small IAS load could be partly linked to reduced opportunities to invade similar acclimatable environments while also having low human density populations to favour distant propagule dispersal. In contrast, we observe the high number of aliens generated per area within a comparatively poorer PD in the case of Australasia. This could be explained by this being a region with a high degree of isolation but with biomes of strong climatic similarity to other temperate parts of the globe. The IAS pattern can be mirrored in the cases of other regional evolutionary factories within Fabaceae and Myrtaceae, which show high numbers of invasive *Acacia* spp. and *Eucalyptus* spp. originated from Australia^39^. In this context, the observed role of climate variables in influencing phylogenetic relatedness of forest assemblages^40^ consistently leads us to question how exactly phylogenetic relatedness and climatic niche breadth interact to shape IAS capabilities globally. Nonetheless, the role of macroclimatic matching from temperate floristic regions cannot be easily disaggregated from historical factors since human colonization has also mostly originated from temperate regions in recent millennia, with assisted directional pathways such as the introduction of bamboo by European settlers to South Africa^33^ and the dominance of ornamental plants from the “New World” among invasive species present in China^41^. Thus, the complex interactions of a range of physiological and morphological traits behind the observed invasiveness remain difficult to unravel. One possible approach could be integrating structural phylogenetic path analyses^42,43^ which the present study assists towards in defining suitable prior models to evaluate.

### Effect of diversification rates on the invasiveness

Overall, our results support the notion that some evolutionary effects do influence the ratio and phylogenetic signal of IAS globally and between most subclades. In this regard, it must be noted that Poaceae has been defined as one of the families with high diversification rates^44^. Interestingly, our results indicate the higher diversification rates are positively related to the frequency of invasive species within Poaceae clades. These results suggest that greater versatility and evolutionary radiation of a clade promote the emergence of new invasive species. Moreover, often grass species have natural weedy life-history strategies that give them inherent capabilities to withstand and flourish in novel environments of an anthropized world^45^. Yet, the invasive status where the species produce substantially harmful impacts can only be achieved by enduring the local abiotic conditions while also being able to compete with native flora^46^. Our results indicate that, but for a few clumps (e.g. *Bromus* and *Lolium*), there are no clearly conserved successful lineages with more invasive capabilities than others. Rather, the results suggest a high versatility of responses and strategies to restrictive pressures, leading to the emergence of some species, particularly well-equipped to become invasive^8^. In this context, the resilience and adaptability of invasive grasses in arid pastures have been documented^47^.

The invasiveness of grasses was also positively linked with recent diversification patterns. This would point to a new acquisition of traits that promote invasiveness. Nonetheless, much remains to be studied regarding the trait correlates responsible for IAS emergence. In the absence of such insights, correlation studies on trait expression along the Poaceae family offer relevant analytical designs to explore them. For instance, the relative expression of two alternative grass persistence strategies towards overcoming natural fire dynamics (resprouter/seeder) has been explored in relation to fire intensity^48^, offering a useful approach to replicate for the case of potential functional traits (e.g. plant height, leaf size or seed mass)^10^ that could act as correlates of invasiveness.

### Examination of patterns of biogeographical factories of invasive species

**Fig. 4:**
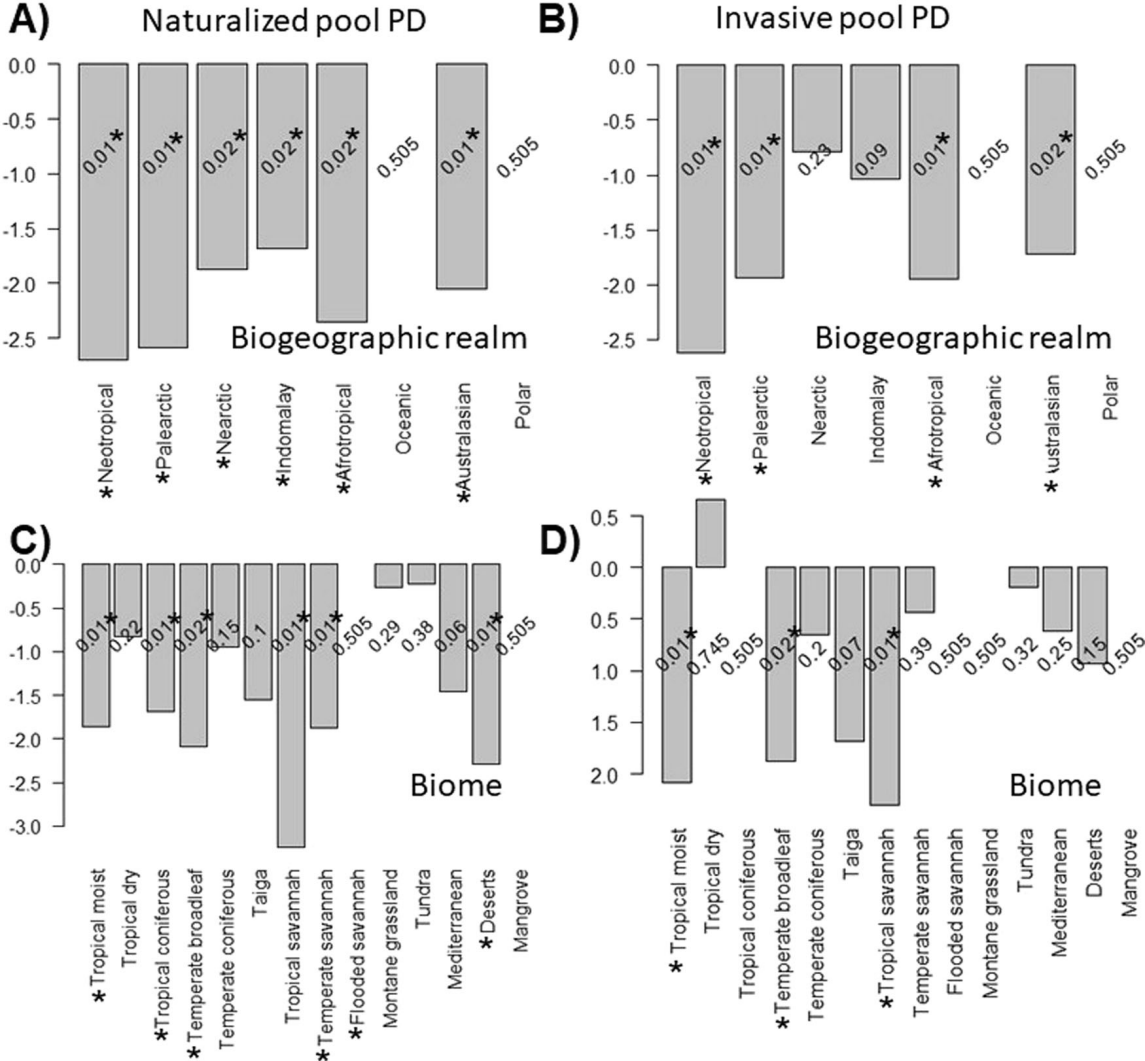
Standardized effect size of phylogenetic diversity (SES PD) controlled by alien species richness. **A**, **B** Indicate the SES scores per realm, respectively, for the naturalization and invasiveness of the factory pool of alien species. **C**, **D** Indicate the SES scores per realm respectively for the naturalization and invasiveness of the regional pool of alien species. *P*-values are indicated in each barplot.

Much work remains to be done to understand reasons for differences in the capacity and structure of different regions to produce (“donate”) invasive grass species as opposed to their susceptibility to invasion by alien species from other regions. In this study, all realms showed a restrictive promotion of certain donor lineages towards acquiring naturalization status (Fig. 4A), although fewer realms retained this feature in the PD structure of invasiveness (Fig. 4B).

Previous studies have identified South Africa as a major donor of alien grasses to other arid savannas in the world^22^ whilst being a recipient of Eurasian ones in anthropically-transformed environments^49^. This landscape-driven disparity could be attributable to resistance to invasion attributes in the native grassland and savanna communities where e.g., their strong adaptability to natural disturbances such as fire regimes can play a significant role in biotic resistance^22^. In contrast, the Brazilian Caatinga is a major recipient of Poaceae invasions^50^ but the Neotropical region is in turn a comparatively lower donor of aliens (Fig. 3, Table 3) under a remarkably high specificity (lowest SES PD, Figs. 4A and 3B). Also interestingly, among biogeographic realms, Australasia is considered a region containing biomes that are highly susceptible to substantial levels of alien species introductions^51^, but also has high levels of exported invasive species per area (Fig 3B2 and 3C2) under a moderately restrictive promotion of alien lineages (Fig. 4A and B). Perhaps the high degree of biogeographical isolation paired with the strong climate similarity of some populated parts of Australia with other Mediterranean and broadleaf biomes could explain this large bidirectional exchange. Furthermore, among biomes, tropical savannas as a donor presented the lowest SES PD values indicating a high degree of phylogenetic selection in the exportation of aliens. In this context, recent evidence indicates that resource-use-efficient invaders outcompete native grass species in Australian tropical savannas^52^. This led us to question the degree of relatedness as well as the comparative PD levels between alien phylodiversity exported (such as the values reported in this study) against the phylodiversity load and structure of received invader counterparts. Examination of import/export matches and mismatches from an evolutionary perspective would provide complementary insights towards further identifying the observed regional patterns of donors and recipients of aliens on Earth^22^, a question that merits additional future research.

In conclusion, in the future scenarios of further global environmental change, those taxa with higher adaptability to disturbed biotic and abiotic regimes would be expected to become more dominant. The high naturalized and invasive species load of Poaceae suggests that further expansion, diversity and distributions of grasses worldwide can be expected in an increasingly anthropically disturbed world. A priority for future research is to explore the latent invasive potential among new alien species that may arise as levels of global change intensify, and the extent to which phylogenetic ancestry provides clues that can be applied to guide predictions. Such evolutionary insights could feed into more informed conservation policies involving pre-emptive biosecurity risk assessments across all taxa.

A moderate degree of phylogenetic conservatism of the invasive character within Poaceae was observed; this suggests that some moderately conserved traits explain the observed phylogenetic signal beyond a purely random evolution caused by the coexistence of highly labile traits. We also observed that the diversification rates influence the emergence of invasive species, suggesting that invasiveness is favoured by recent evolutionary radiations. In turn, the observed variation of the biogeographic patterns of invasive species production also indicates an effect from spatial filters. Biogeographic regions show the disparity in the relative invasive species load but also in the relativized PD levels. Remarkably, some biogeographic realms and biomes showed strong phylodiversity contraptions whereas others act as factories that present high alien diversity.

This study has demonstrated that both evolutionary and biogeographical factors play a significant role in the naturalized/invasive status load and likelihood, and they should be either controlled or accounted for in any structured global meta-analyses of invasive traits. These questions are crucial for alien biosecurity management so as to understand and mitigate present and future biological invasions and the resulting biotic homogenization processes that impact biodiversity conservation.

## Methods

### Data compilation

We use the term “naturalized alien species” (NAS) to refer to any non-native species with established (naturalized) populations. For this categorization, we used the GloNAF compendium^29^. We define “invasive alien species” (IAS) as those species that are regionally expanding or that already occupy an extensive area within any non-native domains, often also having demonstrated evidence of impacts on the native biodiversity^2,30^. Thus, IAS correspond to those naturalized species that are listed as a threat after being formally assessed as such by expert researchers in publications and/or national and international authorities in repositories such as the Global Invasive Species Database (GISD) and/or national catalogues (Supplementary Table 1). However, we note that the different statuses remain subject to different criteria. Therefore, in this study, we operate with our aggregated compendium of reported IAS (Supplementary Data 1) so as to incorporate all forms/criteria of designation, but also provide complementary analyses for the GloNAF list of naturalized species^29^. In order to be able to perform the evolutionary and biogeographical analyses, the status dataset of alien and control species was limited to all grass species present in the phylogenetic tree from a publicly available plant megaphylogeny^28^, containing 1319 resolved species for the Poaceae family.

The initial list of IAS within Poaceae in Global Invasive Species Database (http://www.issg.org/database) present in the phylogeny was retrieved in January 2020. Different national catalogues and published compendiums across the globe were also consulted, thus adding extra invasive species listed in their latest reports. A complementary bibliographic search was conducted with the terms “Poaceae” and “Invasive” on the title and/or abstract of publications registered in the ISI Web of Science (WoS). A total of 450 related publications were retrieved from the WoS repository by January 2021. From the read of these publications, additional IAS described by regional and national studies were added to our IAS list. A total of 25 reportedly invasive species were absent in the GloNAF naturalization list indicating different criteria in the assignation of the alien status and/or newer additions. Thus, these designations by national agencies and researchers were kept for consistency in the inclusive compilation of the invasive species catalogue.

### Evolutionary structure analyses

All statistical analyses were performed in R software v3.5 (Core Team 2018). The Poaceae species tree was pruned to our different lists of grasses with the R package “ape” 5.0^53^. First, we analysed the evolutionary clumping (degree of species relatedness) of the invasive status among Poaceae plants using the PhyloD binary trait estimator under the “caper” R package^21^. This package provides comparative analyses using generalized least square methods. Values close to 1 indicate low to no phylogenetic signal (characters are labile), and values around 0 suggest a Brownian/Neutral character evolution with a tendency for close relatives to behave similarly (evolutionary clumping). PhyloD estimations were conducted for the whole Poaceae family, and for four monophyletic partitions; the Pooideae, Bambusoideae and Oryzoideae subfamilies (all from the BOP clade) and the PACMAD clade (containing Aristidoideae, Panicoideae, Chloridoideae, Danthonioideae, Arundinoideae, and Micrairoideae subfamilies). Basal groups were not analysed individually due to the absence of IAS contained in the present phylogeny.

Ancestral reconstructions of invasiveness were computed next with the R package “phytools”^54^. The ancestral character estimation was performed with fitER function as a re-rooting method for discrete characters by fitting a continuous-time Markov chain model—typically referred to as the Mk model for sequence evolution. The function make.simmap was used to calculate the densities map under 100 simulations. To better visualize the results, a 0.95 lambda transformation of the tree tips was applied. Untransformed and transformed trees (lambda 0.90) for the ancestral reconstruction of both the invasive and naturalized character are provided in the supplementary material (Supplementary Figs. 1–4).

Finally, we computed the diversification rates of the species using Bayesian analysis of macroevolutionary mixtures—BAMM^55^, and analysed the relationship of diversification rates (independent variable) to invasiveness (non-invasive, 0 vs. invasive, 1; dependent variable) using phylogenetic logistic regression^56^ from the R package “phylolm”^57^, assuming a binary response and logit link. For visual representation and comparison purposes, mean phylogenetic diversification values are then displayed per monophyletic clades (by merging paraphyletic complexes of genera with intermixed species as single clades) with either absence (none) or presence (one or more) of invasive species (see Supplementary Table 2 for the ordination).

### Biogeographic phylodiversity analyses

An information criterion was used to allocate the origin of species to the primary regions of occurrence (biomes and floristic realms)^58^. This attribution was done from the highest number of Global Biodiversity Information Facility (GBIF) records (mode). The use of such broad ranges is meant to minimize spatial errors and biases since we still face substantial uncertainties in the designation of the origin floristic region of species at finer resolutions.

Importantly, while PhyloD analyses indicate evolutionary clumping levels irrespective of assemblage composition, the phylogenetic diversity (PD) metrices indicate the broad range of evolutionary variation for a given species assemblage. The Faith’s PD of alien communities per floristic region was calculated with the “picante” R package^59^. In our study, the PD represents a comparative measurement of phylogenetic breadth for each regional invasive grass assemblage within Poaceae. However, due to its additive nature this metric alone offers limited information as it is highly dependent on the species pool size (escalates with species richness volume). Thus, to comparatively evaluate phylodiversity levels across regions we controlled this effect by examining standardized effect sizes (SES) of the PD. This operation was performed by testing the observed levels of invasive species phylodiversity of each region against the expected null diversity levels of the global dataset of all Poaceae species. This null PD score was obtained as the average of a set of random populations (100 bootstrap replicates) from the total Poaceae diversity, respectively, constructed at the given invasive species richness volume per region tested. The SES PD analysis evaluates the degree of phylogenetic diversity in the regional pools of IAS. Specifically, the direct comparison of the effect sizes allows us to identify the communities with the lowest SES PD values as the regions with more restricted clade selection of invasive alien species, whereas those with higher SES PD indicate general invasive factories, i.e. areas with a high clade diversity of invasive species.

### Supplementary information

Supplementary Data

Supplementary Tables and Figures

## Data Availability

Source data was retrieved from public repositories and literature. All datasets are available in the supplementary material.

## Code availability

All code used for data processing and analysis are available at https://github.com/LuisRPertierra/IAS-Poaceae-Phylosignal.
