# Supplementary material for: Contrasting patterns in phylogenetic and biogeographic factories of invasive grasses (Poaceae) across the globe
Source: NPJ Biodivers. 2023 May 18;2:11. doi: 10.1038/s44185-023-00016-4 (PMC11332090; doi:10.1038/s44185-023-00016-4)

Supplementary Table 1. List of repositories of global/national/regional catalogues of invasive species

Database/Ref. Sources	Region/Extent	Countries	Access Link/Extended Reference
Global Invasive Species Database	Global	All	http://issg.org/database/welcome/aboutGISD.asp
Invasive Species Compendium	Global	All	https://www.cabi.org/isc/
State Noxious Weeds (USA)	North America	USA	https://www.invasive.org/
Canadian Botanical Conservation Network	North America	Canada	http://www.rbg.ca/archive/cbcn/en/projects/invasives/i_herb1.html
Especies Invasoras Exoticas SEMARNAT Mexico	North America	Mexico	https://www.gob.mx/semarnat/documentos/listado-de-plantas
Plantas Invasoras en Cuba	Central America	Cuba	Jardín Botanico Nacional. Bissea (9) SpecialIssue 2
Naturalization and Invasion of Alien plants in Puerto Rico	Central America	Puerto Rico, Virgin Islands	Sandoval & Acevedo Rodriguez (2015) Biological Invasions
Inter-American Biodiversity Information Network (Brazil)	South America	Brazil	http://i3n.institutohorus.org.br/www/
Plant Invasions in Chile	South America	Chile	Invasive Species in a Changing World Arroyo et al. 2000 Eds. Hobbs
Invasive Alien Species (EU)	Europe	European	http://ec.europa.eu/environment/nature/invasivealien/index_en.htm
Handbook of Alien Species In Europe DAISIE (EU)	Europe	European	http://www.europe-aliens.org/
Weeds of National Significance	Oceania	Australia	http://www.environment.gov.au/biodiversity/invasive/weeds/weeds/lists/wons.html
Consolidated list of environmental weeds (NZ)	Oceania	New Zealand	Howell and Clayson (2008)
Invasive Alien Plants in China	Asia	China	Weber et al. (2008) Biological Invasions<br>Fang & Wan (2009) CRC Press
Invasive alien flora of India	Asia	India	Wagh& Jain, 2015<br>Kohli et al. (2009) CRC Press
Invasive Species of Japan	Asia	Japan	https://www.nies.go.jp/biodiversity/invasive/DB/etoc8_plants.html
Alien Invasive Plants List For South Africa	Africa	South Africa	https://www.environment.co.za/weeds-invaders-alien-vegetation/alien-invasive-plants-list-for-south-africa.html

Supplementary Table 2. Poaceae species were arranged in monophyletic clades at the genera level. Clades are named after the dominant genera for each cluster. The list of inclusions of related single species (and excluded from their original source) or entire genera (merging of paraphyletic groups) is provided below. This reclassification results are meant to cope with phylogeny uncertainties for Poaceae from Qian and Jin (2016) but ought not to be taken as a resolved phylogeny.

Anthraxon: Eulalia quadrinervis
Agropyron: Henrardia (Elymus rectisetus, Leymus tianschanicus, Elymus pycnanthus)
Aegylops: Triticum
Agrostis: Polypogon
Altoparadisium: Arthropogon
Aira: Deschampsia flexuosa
Andropogon: Schizachryrium
Apera: (Poa eminens)
Arctagrostis: (Poa saxicola)
Arundinella: Garnotia, Imperata cheesemanii
Axonopus: Opiochloa
Bambusa: Dendrocalamus, Oxytenanthera
Bothriochloa: Dichanthium
Bouteloua: Buchloe, Chondrosum
Brachyaria: Urochloa, Eriochloa, Melinis, Thuarea, (Streptostachys acuminata)
Bromus: (Melica mutica) Boissera squarrosa
Echinopogon:: Calamagrostis, Ammophila, Triplachnne (Agrostis thurbenaria), Briza subaristata Dichelachne, Briza (except Briza Maxima),
Chloris: Lintonia, Enteropogon prieurii
Cortaderia: Lamprothyrsis
Cinna: (Alopecurus myosuroides)
Dichelachne: (
Deschampsia: Helictotrichon versicolor, H. bromoides, H.hookeri. Holcus
Elymus: Dasypyrum villosum, Hystrix patula
Eleusine: Leptopchloa, Dinebra
Leptopchloa: Dinebra
Eragrostis: Ectrosia, Eragostiella brachypilla, Harpachne, Pappophorum bicolor, Enneapogon scaber, Pogonarthria, Cladoraphis
Entolasia: Panicum notatum
Fingerhuthia: Entoplocamia
Festuca: Vulpia, Lolium, Micropyrum
Helictotrichon: Arrhenaterum, Hierochloe occidentalis
Hordeum: Elymus glaucus, Elymus enysii, Hystrix laevis, H. gracilis
Heteropogon: Iseilema, Coix
Imperata: Mischantus ecklonii, Saccharum arundinaceum, Saccharum ravennae
Ixophorus: Panicum bulbosum
Guadua: Chusquea coronalis, Chusquea circinata, Otatea
Hackelochloa: Hemarthria uncinata
Triplasis: Gymnopogon brevifolius
Leptothrium: Gymnopogon ambiguous
Leymus: Hystrix duthiei, Hystrix californica Psathyrostachys, Hordelymus
Litachne: Olyra

Muhlenbergia: Aegopogon, Lycurus, Schedonnardus, Redfieldia, Blepharoneuron (Sporobolus contractus, Eragrostis australasica)
Pennisetum: Cenchrus
Poa: Austrofestuca, Cleistogenes serotina, Millium, Festuca kerguelensis, Pseudoscherochloa rupestris, Agrostis canina, Neuropoa
Panicum: (Alloteropsis semialata), Neurachne
Pseudosasa: Semiarundinaria, Pleiobastus
Paspalum: Anthaenantiopsis
Phaenosperma: Bromus remotiflorus
Pogonatherum: Hemarthria altissima
Phyllostachys: Indocalamus, Oligostachium, Chimonobambusa, Ampelocalamus, Yushania, Fargesia, Drepanostachys
Pentameris: Prionanthium
Rytidosperma: Pyrrhantera
Spartina: Calamovilfa longifolia
Sporobolus: Aristida hygrometrica, Calamovilfa gigantea, Eragrostis japonica, Crypsis
Sorghum: Eulalia aurea, Cleistachne sorghoides, Ischaemum, Dimeria, Capillipendium, Phacelurus, Polytrias, Microstegium
Sorghastrum: Tristachya biseriata
Sasa: Phyllosasa
Stipa: Oryzopsis, Psammochloa, Piptochaetium, Ampelodesmos, Stipagrostis plumosa
Setaria: Paspalidium, Panicum antidotale, Spinifex, Pseudoraphilis
Streptostachys: Alloteropsis ciminita
Trisetum: Koeleria, Gaurdinia, Rostraria
Trachypogon: Sorghastrum incompletum
Triodia: Monodia
Tetrapogon: Harpochloa
Ventennata: Apera interrupta
Zizania: Leersia

Supplementary Figure 1. Ancestral state reconstruction of the naturalised character state among Poaceae clades (untransformed).

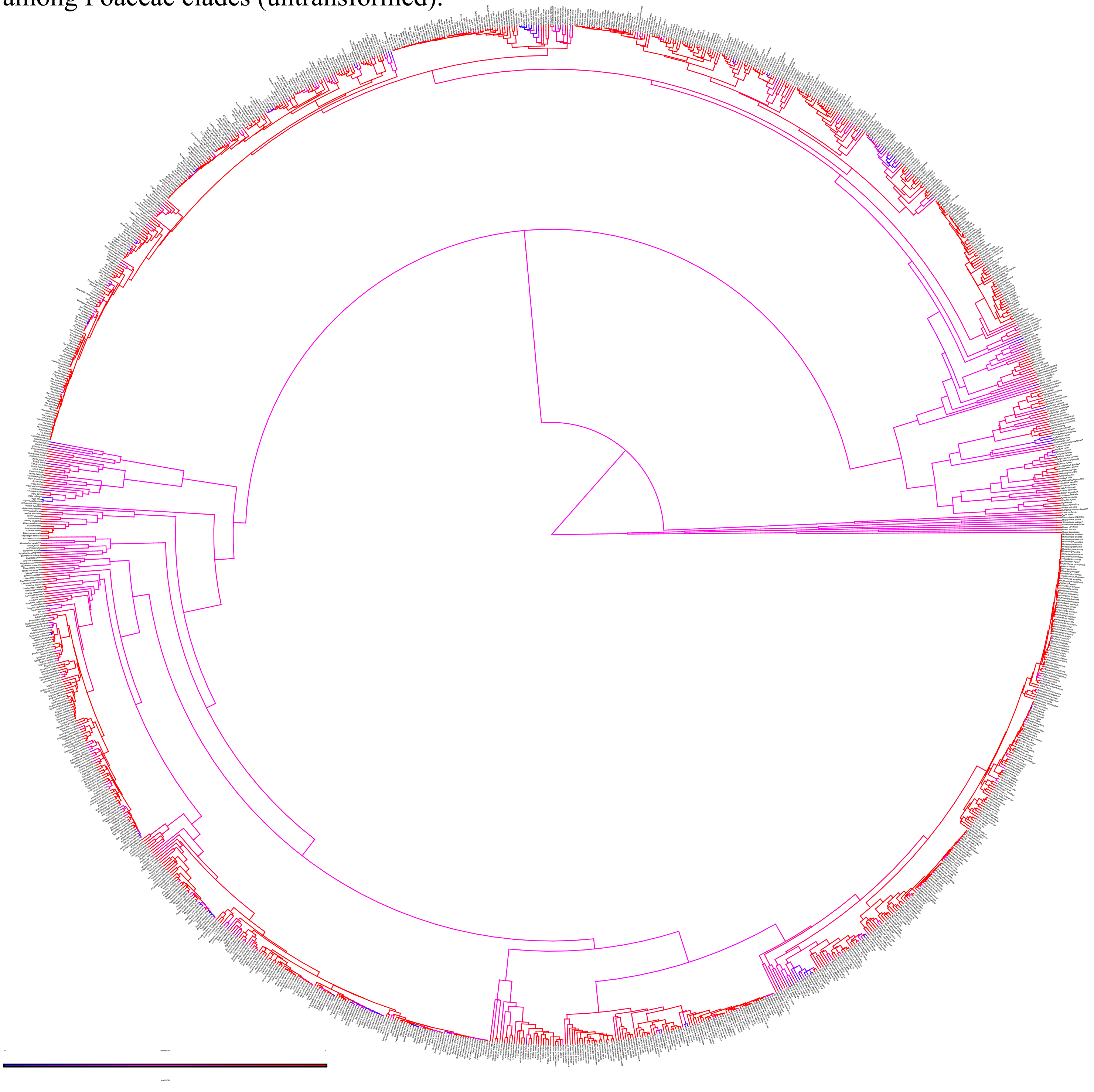

Supplementary Figure 2. Ancestral state reconstruction of the invasive character state among Poaceae clades (untransformed).

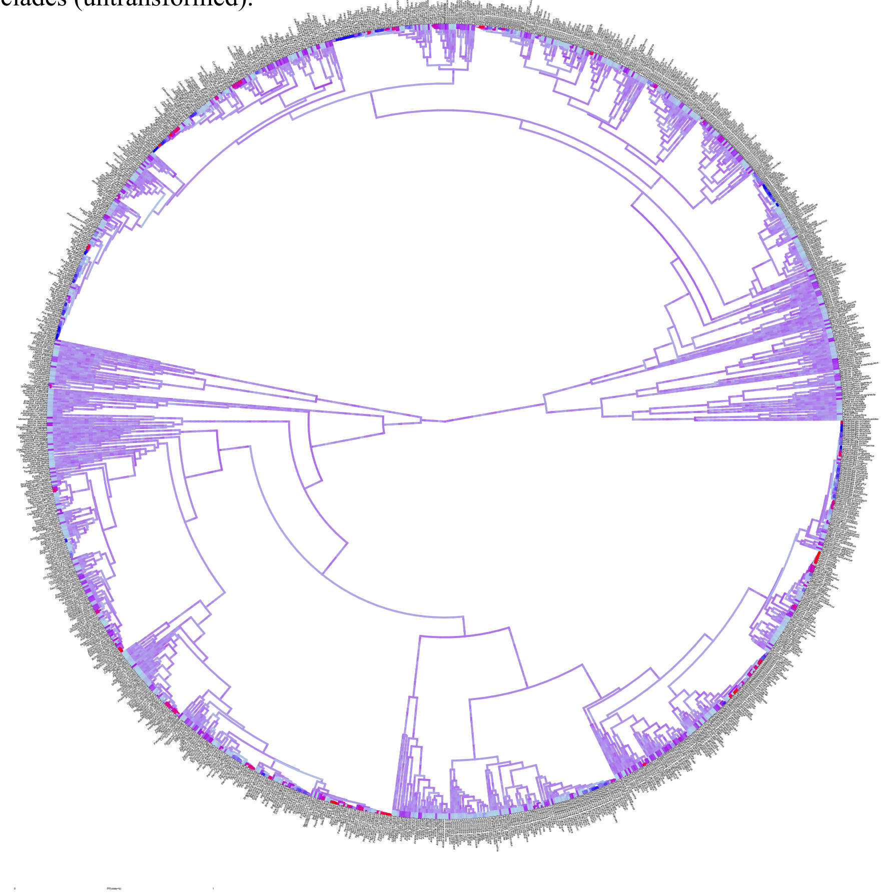

Supplementary Figure 3. Ancestral state reconstruction of the naturalised character status among Poaceae clades with a lambda transformation set to 0.90)

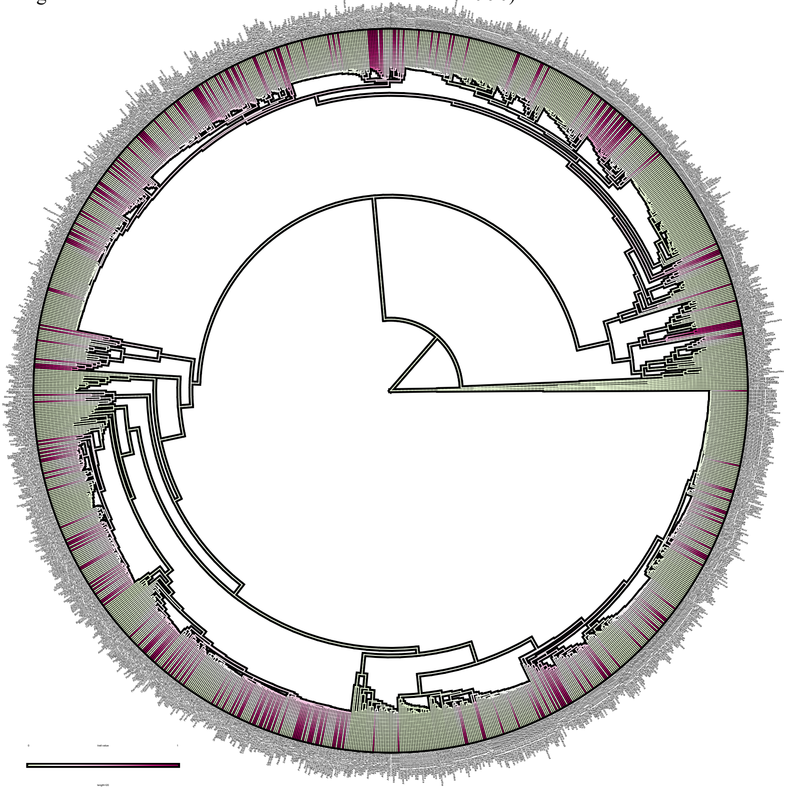

Supplementary Figure 4. Ancestral state reconstruction of the invasive character state among Poaceae clades with a lambda transformation set to 0.90.

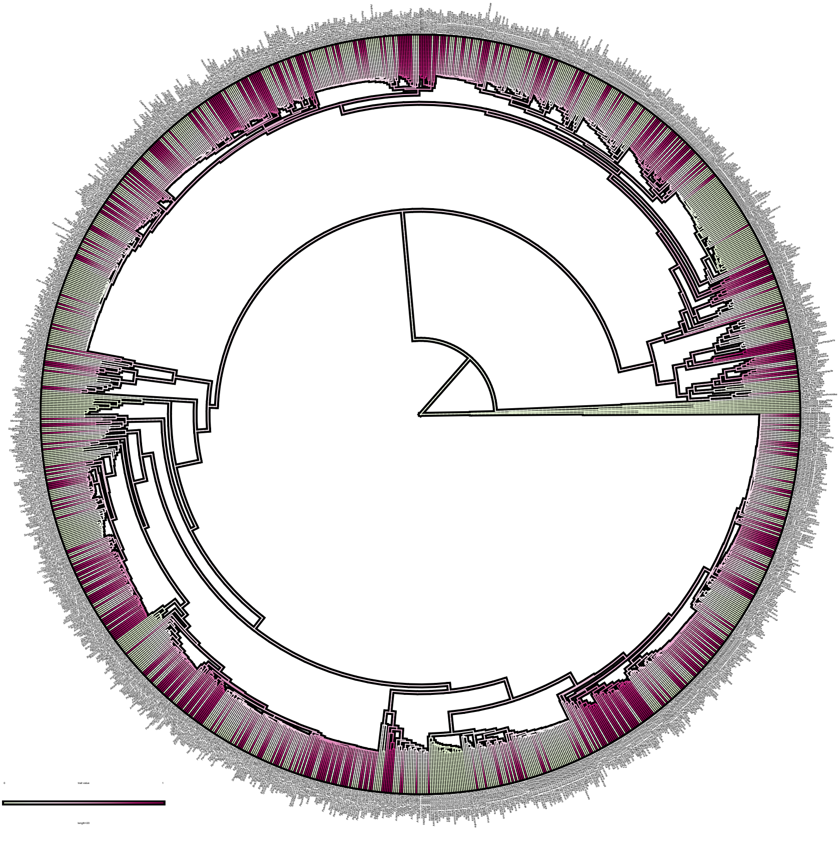

Supplement: Supplementary file 2 — Supplementary Tables and Figures [file 44185_2023_16_MOESM2_ESM.pdf]
